# Omics-Based Strategies in Precision Medicine: Toward a Paradigm Shift in Inborn Errors of Metabolism Investigations

**DOI:** 10.3390/ijms17091555

**Published:** 2016-09-14

**Authors:** Abdellah Tebani, Carlos Afonso, Stéphane Marret, Soumeya Bekri

**Affiliations:** 1 Department of Metabolic Biochemistry, Rouen University Hospital, 76031 Rouen, France; abdellah.tebani@chu-rouen.fr; 2 Normandie University, UNIROUEN, INSERM, CHU Rouen, Laboratoire NeoVasc ERI28, 76000 Rouen, France; stephane.marret@chu-rouen.fr; 3 Normandie University, UNIROUEN, INSA Rouen, CNRS, COBRA, 76000 Rouen, France; carlos.afonso@univ-rouen.fr; 4 Department of Neonatal Pediatrics, Intensive Care and Neuropediatrics, Rouen University Hospital, 76031 Rouen, France

**Keywords:** omics, next-generation sequencing, mass spectrometry, machine learning, chemometrics, data integration, bioinformatics, biomarkers, inborn errors of metabolism, precision medicine

## Abstract

The rise of technologies that simultaneously measure thousands of data points represents the heart of systems biology. These technologies have had a huge impact on the discovery of next-generation diagnostics, biomarkers, and drugs in the precision medicine era. Systems biology aims to achieve systemic exploration of complex interactions in biological systems. Driven by high-throughput omics technologies and the computational surge, it enables multi-scale and insightful overviews of cells, organisms, and populations. Precision medicine capitalizes on these conceptual and technological advancements and stands on two main pillars: data generation and data modeling. High-throughput omics technologies allow the retrieval of comprehensive and holistic biological information, whereas computational capabilities enable high-dimensional data modeling and, therefore, accessible and user-friendly visualization. Furthermore, bioinformatics has enabled comprehensive multi-omics and clinical data integration for insightful interpretation. Despite their promise, the translation of these technologies into clinically actionable tools has been slow. In this review, we present state-of-the-art multi-omics data analysis strategies in a clinical context. The challenges of omics-based biomarker translation are discussed. Perspectives regarding the use of multi-omics approaches for inborn errors of metabolism (IEM) are presented by introducing a new paradigm shift in addressing IEM investigations in the post-genomic era.

## 1. Introduction

Precision medicine (PM) is a disruptive concept that takes into account both individual variability and population characteristics to provide personalized care; this approach widens biological knowledge and explores the great diversity of individuals [1]. PM comprises the customization of healthcare for an individual on the basis of measurements obtained at the individual level. However, it also uses the data and learning retrieved from the rest of the population. Hence, PM relies on both biological individuality and population knowledge to provide tailored healthcare. One of the goals of PM is to use the ever-growing understanding of biology to provide patients with accurate and personalized interventions. All PM strategies include the use of decision-making processes based on biomarker-driven approaches. Genes, gene expression products (i.e., transcripts and proteins), and metabolites are the main biomarker families. Given this molecular diversity of biomarkers, the increase in high-throughput omics technologies offers an amazing opportunity to capture the whole picture of biological systems in a hypothesis-free and unbiased mode. These global strategies are, conceptually, clearly disruptive compared to the current ones, which are mainly hypothesis-driven and, thus, intrinsically reductionist. Holistic investigative methods need to be applied to multiple levels of biological information to deeply understand disease processes.

**Figure 1:**
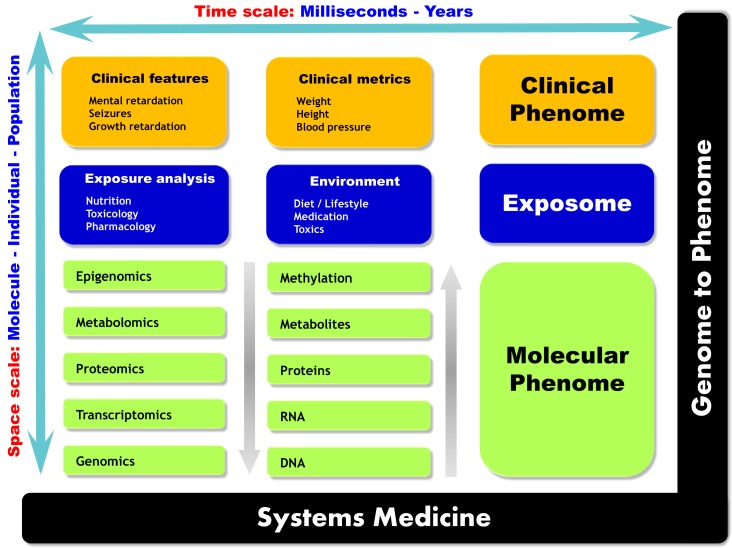
Multi-scale biology overview of systems medicine. Three main drivers define phenotype: (i) the molecular phenome, which is defined by the underlying molecular supports of biological information. The different omics strategies enable to interrogate these supports for information retrieval; (ii) environmental effects spanning from exposures to toxic substances or drugs to diet define the exposome; and (iii) the different clinical metrics used to define the clinical phenome. These different biological and clinical metrics should be approached in a multi-dimensional fashion and should take into account the inherent spatial and temporal scales of both measurement technologies and disease dynamics from the molecular to the population level.

The prediction of normal and pathological states in patients is based on a dynamic understanding of gene–environment interactions on individual and population scales [2]. The new concept of systems medicine relies on global and integrative approaches for patient care. A biological system can be fully understood only if the space and time scales are considered. Figure 1 gives an overview of the multi-scale perspective of systems medicine.

For centuries, biological sciences independently addressed the different parts of life systems and physicians viewed and addressed diseases. Global information retrieval allows contextual pathophysiology understanding of the disease for better diagnosis and treatment [2,3]. Structure, organization, and function descriptions should be considered for a complete understanding of a given biological system. The structure involves basic biomolecules (genes, gene expression products, proteins, and metabolites). The topological connections between these molecules define the organization. The function reflects how the system evolves with regard to metabolic fluxes and environmental stimuli [4,5].

Inborn errors of metabolism (IEM) are an appealing model for systems medicine because the disrupted pathways underlying these diseases have been described at least to some extent. IEM clinical presentations are often non-specific; therefore, appropriate laboratory tests are pivotal for making a diagnosis [6]. However, the widespread routine laboratory diagnosis strategies are mainly represented by sequential investigation assays. This approach is slow and lacks an integrated overview of the generated data. For faster and effective IEM screening and diagnosis, a paradigm shift in investigation strategies is urgently needed. A part of the answer may be found in the new field of systems medicine that capitalizes on omics surge, bioinformatics, and computational advancements to translate the huge amount of data generated by high-throughput omics technologies into effective clinically actionable tools to aid medical decision-making.

In this review, omics technologies that allow holistic biological information retrieval are described. Furthermore, the huge potential of multi-omics data integration strategies within the clinical context is described, as is its role as a key driver for the clinical actionability of omics-based biomarkers. Challenges facing their clinical implementation are then discussed. There is a focus on the relevance of the use of these strategies in IEM.

## 2. Omics Revolution in Translational and Clinical Contexts

Since the discovery of the DNA structure [7], great advances have been made in understanding genome complexity; these advances have led to sequencing the whole human genome using international endeavors such as the Human Genome Project [8]. Genomics approaches have been widely adopted in biomedical research and have successfully identified the genes and genetic loci involved in the development of human diseases [9,10,11]. These findings revealed the complexity of biological systems and provided insights for new approaches to disease diagnosis, treatment, and prevention [12,13,14,15]. Additionally, other high-throughput omics technologies have been developed to measure other biomolecules, such as epigenomics for epigenetic markers, proteomics for proteins and peptides, and metabolomics for low-molecular-weight metabolites. High-throughput analytical methods allow us to study a large number of omics markers simultaneously. In many ways, omics association studies are similar because they search for omics biomarkers connected with phenotype by unbiased ome-wide screening. Given the uneven maturity of the different omics technologies, genomics seems to be the closest next-generation sequencing (NGS)-based technology introduced into the clinic compared to transcriptomics and epigenomics, which are still promising. Regarding mass spectrometry (MS)-based omics, metabolomics seems to be closer than proteomics to being introduced into clinical practice because metabolite analyses using MS are already routinely adopted in clinical laboratories for drug monitoring and IEM screening. In this review, we mainly focus on mature omics technologies that are actively involved in clinical practice to achieve the promise of PM. However, an overview is also given regarding all omics methods.

### 2.1. Omics Technologies

#### 2.1.1. High-Throughput Sequencing (HTS) Technologies

Next-generation sequencing (NGS) techniques using a massive parallel sequencing strategy have profoundly changed the clinical genomic landscape. HTS techniques can be classified according to their applications for investigating genomes, epigenomes, or transcriptome. NGS-based strategies that could be used in medical diagnostics vary according to the size of the interrogated genome. These strategies include capturing the few protein-coding regions of a selected panel of genes (tens to hundreds), sequencing of the entire genetic code of a person, which is called whole-genome sequencing (WGS), and sequencing parts of the genome that contain exonic regions, which is called whole-exome sequencing (WES). WGS and WES are used to discover variants associated with a cell function or a disease [16,17,18]. However, NGS-based transcriptome analysis (RNA-seq) [19] entails quantitative gene expression profiling, whereas epigenomic methods focus on chromatin structure [20].

##### Genomics

The genome is the complete set of DNA of an organism. This genetic material is mainly found in the nucleus of the human cell (nuclear DNA). Mitochondria contain their own genome (mitochondrial DNA). Fredrick Sanger described the chain-termination strategy to replicate a nucleotide sequence of a DNA fragment (500–1000 bases) [21]. Sanger used chemically modified nucleotide bases and radioactive labeling, along with DNA polymerase, primers, chain-terminating nucleotides, and electrophoresis. Since then, sequencing chemistries evolved using fluorophore-labeled dideoxynucleotides and thermostable DNA polymerases allowed cycled sequencing. Electrophoresis automation and laser detection enhanced the sensitivity of this method [22,23,24]. The replicated DNA fragments produce signals (electropherogram peaks) related to the nucleotide sequence. Thereafter, these reads undergo an alignment step with a reference genome to identify variants and define their genomic origin. Of note, the Sanger method is still considered the standard method for DNA sequencing accuracy of approximately 1 in 10,000 bases. The first human genome sequence was achieved in 2001 [25,26]. The genome sequences of several model organisms were determined soon thereafter. These endeavors were accomplished with Sanger DNA sequencing, which involves high costs and low throughput. These drawbacks limited the potential of DNA sequencing for healthcare translation. Several HTS technologies were developed soon after the release of the human genome sequence [27] and high-throughput analysis became widely available for genomics. NGS-based platforms provide the ability to replicate, in parallel, many overlapping short DNA fragments (50–500) derived from already prepared libraries. There are different innovative approaches to the special separation of fragments on arrays or beads [8,28,29]. Simultaneous DNA replication of each fragment during the reaction cycles produces billions of short elongations of the DNA sequence. These short stretches are called reads. Hence, each base is synthesized several times. The lowest number of times that each base being monitored is incorporated into an overlapping fragment is called depth of coverage. At the end of the cycle, all the short reads are assembled according to a reference sequence that allows for reconstruction of the original sequence, ranging from a small exon to an entire genome. Innovative high-throughput NGS-based methods have the ability to conserve the genome information and the redundancy of the sequenced genome through their depth of coverage. Different commercial HTS platforms exist. These platforms differ mainly in their sequencing strategies (ligation versus synthesis), amplification by polymerase chain reaction (PCR) of the DNA fragments (flow cell bridge PCR versus bead emulsion PCR), and finally in their adopted targeted approach (PCR amplification versus hybrid capture) [8].

HTS are powerful technologies for personal genome and transcriptome sequencing [8]. Variants can only be interpreted with a good clinical history, family history, and physical examination. These preliminary steps allow physicians to assess whether there are similar or related phenotypes in other family members; if so, then the inheritance pattern can be evaluated. However, clinical validity is the most challenging aspect of NGS. According to the size of the interrogated genomic information, three strategies could be used for diagnosis purposes. Targeted gene sequencing panels are useful tools for analyzing specific genes in a given clinical condition and are widely used in current clinical practice [14,15]. WES focuses on the more functional and informative part of the genome and is being adopted for genetic studies of IEM for gene identification and clinical diagnosis [10,11,30,31]. This approach might shortly replace targeted approaches. WGS provides a unique window to investigate genetic or somatic variations, thus leading to new avenues for exploration of normal and disease phenotypes. However, the inherent data management and interpretation issues hamper its clinical implementation [32]. From a clinical perspective, comparing different genomic diagnostics approaches is of great interest but requires standard and adopted metrics [33]. Sanger sequencing is the gold standard and allows confident calling of genotypes. Because non-inferiority is a prerequisite for clinical adoption of any new medical innovation, Goldfeder et al. recently proposed an interesting metric to quantify the clinical grade reporting standard of sequencing technologies [34].

##### Epigenomics

Chemical modifications of DNA, histones, non-histone chromatin proteins, and nuclear RNA define the epigenome. These changes affect gene expression without altering the base sequence. Epigenetics usually refers to the structural adaptation of chromosomal regions. These epigenetic marks may be transient or inherited through cell division [35]. They are due to environmental exposures at various developmental stages throughout the life span [36]. The four main actors of epigenetic machinery include DNA methylation, histone modification, microRNA (miRNA) expression and processing, and chromatin condensation [37,38]. Epigenomic modifications depend on spatial and time-related factors. Therefore, they can be tissue-specific in response to environmental or disease-related modifiers. These modifications could regulate gene expression and, thus, affect cell homeostasis. Comprehensive mapping of epigenetic makeup in many cell types and tissues has been reported [39]. Different strategies have been developed to assess the epigenome [20]. Epigenomics methods generally focus on chromatin structure and include histone modification ChIP-seq (chromatin immunoprecipitation sequencing), thus allowing the identification of DNA-associated protein-binding sites [40]. DNase-seq combines DNase I digestion of chromatin with HTS to identify regulatory regions of the genome [41]. DNA methylation [42] and ATAC-seq (assay for transposase-accessible chromatin sequencing) allow the mapping of chromatin accessibility genome-wide [43]. For more technical details, the reader may refer to a recent review [44]. Recently, an epigenome-wide study suggested that interindividual variations in high-density lipoprotein (HDL) particle metabolism rely on epigenome modifications [45].

##### Transcriptomics

The gene expression pattern in a cell/tissue can broadly reflect its functional state. The transcriptome is the complete set of RNA transcripts, including ribosomal RNA (rRNA), messenger RNA (mRNA) that represents only 1.5 to 2 percent of the transcriptome, transfer RNA (tRNA), miRNA, and other non-coding RNA (ncRNA). Quantitative analyses of the transcriptome can be performed with either microarrays (Chips) or RNA sequencing (RNAseq). Microarrays are based on specific hybridization of RNA transcripts to DNA probes, and HTS-based expression profiling by RNA-seq allows comprehensive qualitative and quantitative mapping of all transcripts [19]. The massively parallel capabilities of HTS have dramatically widened the transcriptional landscape with small quantities of total RNA [46]. Transcriptome-based studies have been applied to some IEM such as McArdle disease [47], Hunter disease [48], lysinuric protein intolerance [49], Lesch–Nyhan disease [50], and Niemann–Pick C disease [51].

#### 2.1.2. Mass Spectrometry-Based Omics

MS analyzers are instruments that weigh molecules and separate them according to their mass-to-charge ratios. There are several MS analyzers with different analytical technologies and, thus, various performance levels regarding resolution, accuracy, throughput and chemical coverage. MS analysis could be semi-quantitative in an untargeted fashion using high-resolution MS instruments or quantitative through targeted analysis using tandem MS [52,53,54]. MS instruments could be combined with separation methods such as liquid or gas chromatography, capillary electrophoresis, or ion mobility. These combinations aim to enhance the dynamic range, sensitivity, specificity, and chemical coverage [55,56]. Given the chemical diversity of proteins and metabolites and the high sensitivity of this technology, MS has proven its superiority in metabolomics and proteomics.

##### Proteomics

The proteome consists of all the proteins expressed by a biological system [57]. Posttranslational modifications rely on a highly specialized enzymatic arsenal specific to each cellular type, which leads to the generation of different proteomes from the same genome. These modifications add layers in proteome complexity and, thus, broaden their functionalities [58]. Hence, proteins exhibit different conformation, localization, and interactions depending on space and time factors. The development of proteomics assays is triggered by these complexity challenges. The proteome can mainly be analyzed using MS or protein microarrays [53,59]. However, MS and protein separation allow rapid and accurate detection of hundreds of human proteins and peptides from a small amount of body fluid or tissue [59,60,61]. Recent studies showed promising results using proteome analysis to explore cystinuria [62], mucopolysaccharidoses [63], and liver mitochondrial functions [64]. Despite increasing analytical performances, proteomics has not been used in routine clinical laboratory practice [65].

##### Metabolomics

The idea behind metabolomics or metabolic profiling has been empirically used in the past; for example, urine organoleptic characteristics (taste, odor, or color) aided in the diagnosis of medical conditions [66]. The metabolome is defined as the set of metabolites present in a given biological system, fluid, cell, or tissue at a given time [67]. Metabolomics is an omics approach based on biochemical characterizations of the metabolites and their fluctuations related to internal (genetic) and external factors (environment) [68]. The metabolomics approach has been applied in many disease studies [69,70]. MS and nuclear magnetic resonance (NMR) are the main analytical techniques used in metabolomics [71]. However, MS is already adopted in clinical laboratories. New advances in analytical technologies such as ion mobility spectrometry (IMS) combined with high-resolution MS have allowed better coverage of the metabolome [56]. Because IEM are related to metabolism disruption, metabolomics is indicated in assessing these diseases. The future of IEM diagnoses relies on simultaneous quantitative metabolic profiling of many metabolites in biological fluids. Targeted MS-based metabolomics is already widely used and implemented in IEM newborn screening national programs worldwide [72]. Untargeted approaches have also been tested and have shown promising results [73,74]. An integrated strategy for IEM assessment using both targeted and untargeted approaches has been recently proposed by Miller et al. This strategy provides useful and actionable diagnostic information for IEM. The authors have successfully diagnosed 21 IEM disorders using plasma metabolite measurements through metabolomics [75]. Aygen et al. performed a multi-center clinical study in 14 clinical centers in Turkey using NMR-based platforms. The urine samples of 989 neonates were analyzed. A set of specific metabolites that varies in patients compared with healthy individuals was characterized and predictive models were developed. Furthermore, a reference NMR database has been built [74]. For a deeper overview of the potential of metabolomics in IEM investigations, refer to a recent comprehensive review reporting underlying metabolic profiling technologies with limits and advantages and their applications in IEM [76].

#### 2.1.3. Phenomics

Phenome is a term that describes the measurable physical and chemical outcomes of the interactions between genes and the environment that are experienced by individuals and influence their phenotypes [77]. Hence, phenotypes could be retrieved through precise, quantitative analysis [78]. Phenomics, which is a branch of science that explores the basis of how our genes respond to environmental changes, is an emerging and powerful approach to revealing important human attributes at the molecular level. It aims to explain how we adapt and why we are affected by diseases [79]. In other words, phenomics approaches capture our personalized experience with our environment [80,81,82,83,84].

Two main pillars build phenomics: deep phenotyping (DP) and phenomics analysis (PA). DP refers to a strategic and comprehensive approach to data acquisition that includes clinical assessment, laboratory analyses, pathology, and imaging. PA involves the evaluation of patterns and relationships between individuals with related phenotypes and/or between genotype–phenotype associations. PA relies on both clinical data and high-dimensional data integration [85], analysis, and visualization [81,86,87].

In a recent work, Kochinke et al. provided a curated database of 746 currently known genes involved in intellectual disability (ID). The genes were classified according to ID-associated clinical features. This work allowed systematic insights into the clinical and molecular landscapes of ID disorders [88]. Kim et al. introduced the integrative phenotyping framework (iPF) for disease subtype identification. This solution allows accessible visualization of multi-omics data following effective dimension reduction. The strategy has been successfully applied to chronic obstructive lung disease (COPD) [89]. The Monarch initiative is an impressive global endeavor that provides computational tools for genotype–phenotype analysis, genomic diagnostics, and PM across broad areas of disease. Thus, the Monarch initiative illustrates the importance of phenomics [90].

For more details, the reader may refer to a recent review reporting state-of-the art phenome-wide association studies [79].

### 2.2. Multi-Omics Strategies, or When the Whole Is More than the Sum of Its Parts

Although each omics technology is able to measure one family of biomolecules accurately and comprehensively, they are all limited by the functional roles of each type of molecule in a biological system. With the significant advancement of high-throughput technologies and diagnostic techniques described here, the molecular basis of many disorders has been unveiled and their integrative consideration could help solve this issue. However, translation of a patient-specific molecular mechanism into personalized clinical applications remains a challenging task that requires integration of multi-dimensional molecular and clinical data into patient-centric models. For example, family history, clinical history, and physical examination are mandatory for the interpretation of variants and laboratory results. However, in NGS, reporting the result is a very tricky task. NGS test accuracy is at its best when the considered variant in a given gene has been previously associated with the patient’s condition and when a conclusive functional test has revealed the gene’s function abnormalities. Furthermore, few functional studies are available regarding the biological effect of individual variants. This largely impedes effective and comprehensive interpretation of NGS data. In this regard, PM combining multilayer molecular information and specific clinical phenotypes for a given patient may be an answer to this limit [1]. Applying the PM concept to omics and clinical data is a challenging and exciting task. This integrative view of disease modeling is an emerging knowledge-based paradigm in translational and clinical research that capitalizes on the ever-growing power of computational methods to collect, store, integrate, model, and interpret curated disease information across multi-scale biology from molecules to phenotypes [85,91]. With the tremendous amount of available biological and clinical data, the development of appropriate data mining tools is mandatory to extract the hidden information, thereby allowing its translation into actionable clinical tools [91,92]. As technologies keep evolving and datasets grow in volume, velocity, and variety in the big data era, a strong informatics infrastructure will be essential to embrace the PM promise of improved healthcare derived from personal data. Different computational solutions using machine learning and dimension reduction methods have been developed for omics integration [93]. Recent studies have shown the potential of multi-omics studies to provide insightful biological inferences [64,94,95,96,97,98,99] and to help determine definitive diagnoses in the IEM field [10,11].

### 2.3. Issues and Limitations of Omics Analysis

#### 2.3.1. Technical Limitations

##### Experimental and Analytical Noise

Reproducibility and repeatability are prerequisites for obtaining consistent results [100]. These important validation steps are hampered by the so-called batch effects. In addition, this drawback can be an important confounder in association studies and potentially causes spurious associations unrelated to the outcomes of interest. Multiple technical platforms from different manufacturers are usually available for the same type of omics profiling. For example, multiple versions of microarray and sequencing platforms are available for genomics, transcriptomics, and epigenomics association studies. They usually have different coverage of the sequenced regions [44]. MS platforms for proteomics and metabolomics have different sensitivities and chemical space coverage [61,76]. This is due to the differences in MS analyzer technology in terms of ionization method, resolution power, measurement accuracy, multi-dimensional separation, scan speed, dynamic range, and analysis throughput [55,56,76]. Such technical heterogeneity often makes meta-analysis and data fusion of different omics studies very challenging. Batch effects issues can be handled by using harmonized Standard Operating Procedures (SOPs) [101,102,103,104]. Furthermore, using standard quality control (QC) processes and metrics to normalize intra-laboratory and inter-laboratory omics measurement variations [105,106] and applying consistent statistical correction methods [107,108,109] and appropriate computational tools [110] can address some technical variation issues.

##### Analytical Accuracy and Clinical Relevance

Historically, genomics has tightly evolved along with reference sequence GRCh38 [111]. The genome contains approximately 20,000 protein-coding genes, and these vary enormously, spanning from eight base pairs (a transfer RNA) to millions of base pairs. For a given gene, the exon number spans from one to hundreds. Furthermore, a gene’s GC richness is a great challenge, especially for capture chemistry-based targeted sequencing. This great genome complexity presents challenges for NGS sequencing strategies regarding accuracy, which is the mandatory prerequisite for clinical implementation. For example, given the intrinsic short-reads sequencing strategies of HTS, simple repeats that are shorter than the read could be determined with NGS. However, if the read length is shorter than the repeat stretch, then the size of the repeated region is difficult to define [112]. Another clinically relevant challenge to using short reads is the lack of phase information, which is the parental chromosomal origin. The characterization of compound heterozygosity (two identified variants in the same gene) is challenging and, thus, illustrates this limitation. A variant-calling algorithm solution has been developed to handle such issues [113]. To solve some of these challenges, long-read sequencing strategies such as using either longer molecule barcoding fragments combined with short-read sequencing and in silico assembly [114] or longer molecule direct sequencing may be of interest [115]. Such sequencing strategies may provide a more accurate view of the genome. Chaisson et al. provided seminal evidence for the utility of long-read sequencing to generate high-quality reference genomes. The authors closed euchromatic gaps in the GRCh37 human reference genome using long-read sequencing [115]. Another major drawback of existing NGS strategies is the need for time-consuming library preparation and DNA enrichment. More automation of this step would enhance the workload and turnover and dramatically change the adoption of NGS into the clinical environment, which requires a high standard of accuracy and rapid reporting of results. The use of nanopores is a promising technology that could overcome this limitation by directly sequencing DNA fragments by passing through nanopores using either nanophotonic chambers [116] or a protein nanopore [117].

Regarding MS-based omics, there are still great challenges regarding their widespread use in the clinical environment. For metabolomics, the great drawback is still metabolite identification, particularly for untargeted approaches [118]. Accurate curated spectral repositories are essential to their clinical adoption and compliance with regulatory issues. Furthermore, harmonization of data reporting and data visualization in clinically accessible formats limits their clinical implementations [76]. Proteomic analysis is technically challenging and has major drawbacks due to splice variants and post-translational modifications of divers. The post-translational modifications interfere with DNA and RNA measurements of protein level predictions [119,120]. Better proteomic measurements require unbiased identification and quantification of proteins by direct measurements using methods analyzing their unique structure, mass, and charge with high specificity [106,121,122]. Furthermore, subtle changes in the detection of low-abundance proteins, which are often important in early-stage disease screening, might be affected by MS sensitivity limits. To overcome this limitation, different approaches have been proposed. One approach is immunocapture enrichment of low-abundance proteins prior to their MS detection [123] at the expense of additional steps in the analytical process, which may affect the throughput. The MS-based omics community is aware of these limitations and actively strives to overcome them [54,104,124].

##### Omics Informatics Pipelines in the Clinical Environment

**Figure 2:**
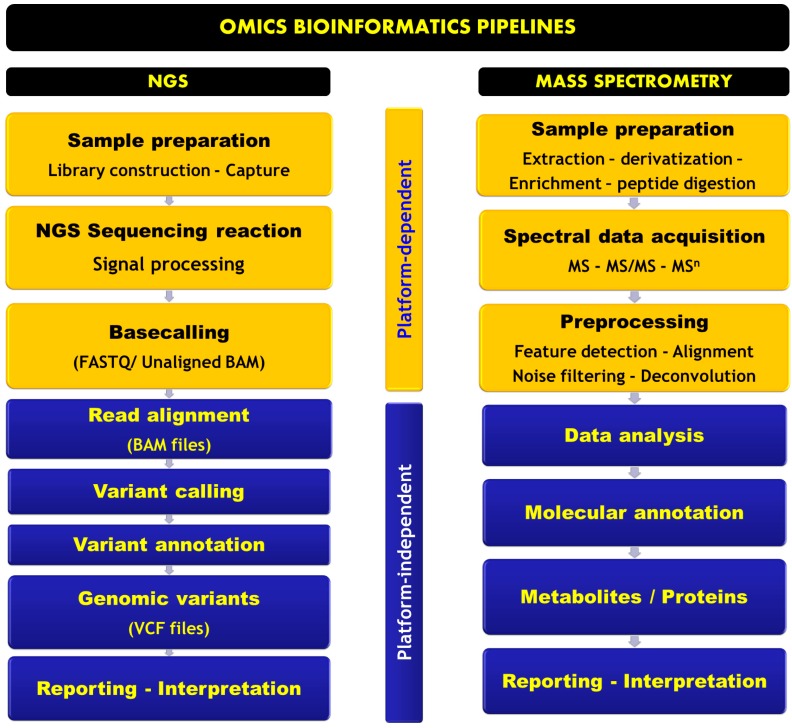
Schematic illustration of bioinformatics pipelines in next-generation sequencing (NGS) and mass spectrometry (MS)-based omics. **Left**: The NGS pipeline comprises library construction and capture, sequencing reaction, and signal processing. Then, a base-calling step is performed to define the unaligned nucleotide sequence. The data are stored in FASTAQ file format containing quality scores. Subsequently, read alignment to a reference sequence is performed, followed by variant calling and annotation. The final output is a list of variants in VCF format for visualization and interpretation; **Right**: MS pipeline starts with sample preparation, depending on the MS instruments and the combined separation method. Data acquisition is performed according to the chosen mode (full scan or tandem MS). Subsequently, a pre-processing step is needed for feature extraction and data cleaning. The result is a list of features that will undergo data analysis, molecular annotation, and identification before biological interpretation. Signal processing is platform-dependent in NGS; however, open source solutions are available for pre-processing MS data.

A bioinformatics pipeline is a sequential series of computationally complex data analysis processes spanning from raw data retrieval to final results output. The series includes processing, data analysis, and interrogation of reference databases. Two main pipelines are discussed here, NGS and MS-based pipelines. Figure 2 represents a schematic overview of both pipelines.

##### NGS Informatics Pipeline

Millions of reads are generated by most common NGS platforms using short reads that overlap either the whole genome (WGS) or a specific region (targeted sequencing). An NGS pipeline includes platform-specific software to generate the sequence derived from the primary instrument signal; this step is called base calling. Subsequently, alignment is performed against a reference human genome sequence of the overlapping reads. Several alignment algorithms have been developed with different performance results regarding sequence variation detection [125,126]. The aligned reads are used as input files for single-nucleotide variant (SNV) detection, copy number variation (CNV), indels, and large rearrangements using open source or commercial tools. This step is called variant calling. Several variant callers are available, such as Atlas [127], MuTect [128], VarScan2 [129], and Genome Analysis Toolkit (GATK) [130]. It should be noted that these variant callers exhibit different performances depending on the platform and variant types used [131]. Thus, using different variant callers for a wider coverage of variants capture is recommended. Further annotation of the detected variant is performed using clinical data, Human Genome Variation Society annotations, genome–phenotype correlation, pathway analysis, and predicted effect (on transcription and translation). The quality and consistency of the interrogated online databases are crucial for this step. To avoid misdiagnoses, interpreting variants should be approached as a dynamic big data problem because these online databases are constantly evolving as disease knowledge evolves [132]. NGS is rapidly making its way into the clinic, and its smooth integration upstream and downstream of a sequencing analysis is becoming an important issue. Informatics challenges facing the implementation of NGS in clinical environments range from data acquisition to data reporting, including data validation, data analytics, data storage, and interoperability with already existing laboratory systems and clinical informatics infrastructures. Sample tracking and workflow management logistics are the core of any clinical grade laboratory; this should also be true for NGS. However, uncommon but important downstream offline steps should be consistently tracked such as nucleic acids extraction, library preparation, sequencing runs along with upstream steps such as bioinformatics analysis, quality assurance documentation, data interpretation, and results reporting. All these steps add complexity and error sources to the workflow. To overcome interoperability problems that face in-house custom solutions, such as fragmenting of the workflow, the ideal informatics solution should be fully integrated with the laboratory information system (LIS) so it is able to track samples from order receipt to results reporting. Of note, the generated NGS data range from 10 GB for WES to 150 GB for WGS. Hence, data storage solutions need to be addressed before implementation. Data analysis challenges not only include the computationally heavy burden of the NGS bioinformatics pipeline but also involve handling the huge amount of background data related to wet laboratory steps, sample meta-data, sample processing and tracking, reports, and QC data. With all the high-dimensional data management issues, NGS clinical implementation should be approached with big data analytics solutions [133].

##### Mass Spectrometry-Based Omics Informatics Pipeline

MS-based processing methods involve four main steps: (i) data acquisition; (ii) data pre-processing; (iii) data analysis using chemometrics; and (iv) identification, network, and pathway analysis [76]. Data files are acquired with proprietary software depending on each platform. Various proprietary data formats have been developed by MS manufacturers to handle MS data, but this raised sharing and processing limits between platforms. To address this problem, open formats have been developed such as netCDF, mzDATA, mzXML, and mzML [134]. Data pre-processing include peak detection, peak alignment, which is a drift time correction step in separated methods (gas chromatography-MS, liquid chromatography-MS, capillary electrophoresis-MS, and ion mobility-MS). During alignment for untargeted analysis, it is crucial to match peaks corresponding to the same analytes in different samples. Subsequently, baseline correction and spectral deconvolution for visualization are performed. Depending on the algorithm used, the order of these steps might be different [135]. The output of these steps is a matrix containing feature concentration or intensity across the different samples. Different output formats such as txt, csv, or an Excel spreadsheet could be used. Subsequently, before data analysis and modeling, different filters, transformations, and normalization methods could be applied to the generated matrix to handle the noise and clean the data. Then, various pattern recognition and machine learning techniques are applied to extract the important features (metabolites or proteins) for the next identification step and pathway and network analysis [76]. MS-based bioinformatics pipeline challenges are the same ones described for big data scaling and interoperability issues with Laboratory Information System (LIS) in a clinical environment. However, some limitations are specific to these platforms in particular, such as sample extraction and/or derivatization, which are offline processes that should be consistently tracked. The metabolite or protein identification steps still lack smooth and streamlined informatics solutions for direct database interrogation. From an informatics perspective, NGS seems to be much more advanced to be included in clinical practice. Therefore, many endeavors are needed to enhance MS informatics infrastructures to a clinical grade [136], and some initiatives have already begun [104,137,138].

#### 2.3.2. Biological Variation

Biological variation is another source of discrepancies in omics studies. Except for genetic profiles being identical across tissues and cell types, all other omics profiles depend on sample type. Tissue and cell-type specificity lead to two important issues in multi-omics approaches: tissue and cell type selection and heterogeneity of tissues. The most accessible specimen in human samples is peripheral blood. Blood-based specimens such as plasma, serum, and leukocytes are commonly used in omics studies. Although the use of blood as a surrogate tissue is sometimes relevant, the biological relevance of blood omics profiles may not be apparent for many human diseases. Using blood-based specimens is a convenient start for searching novel disease-related biomarkers; however, using blood as a surrogate tissue requires cautious validation and interpretation to unravel disease mechanisms [139,140,141]. Furthermore, diet, circadian rhythm, and drugs may interfere. Another issue is cell heterogeneity; a tissue sample always involves several cell types, with each having a unique omics profile. Depending on the location of a tissue sample or the individual physiological condition, the proportions of the different cell types can change substantially. Statistical methods have been developed to adjust for potential confounding effects due to cell-type heterogeneity [142,143,144]. However, measuring the omics profile of each purified cell type is an ideal solution that could directly infer the molecular mechanism of a disease [145].

## 3. Omics and Biomarkers: From Bench to Bedside

### 3.1. Definitions

A biomarker has been defined as a trait that can be objectively measured and evaluated; therefore, it can be used as an indicator of biological processes (normal versus disease) or of pharmacologic response upon a therapeutic intervention [146]. The FDA defined a biomarker as a measurable endpoint that may be used as an indicator of a disease or physiological state of an organism. According to these definitions, several indicators may be included, such as imaging-based or laboratory-measured biomarkers [147]. The Institute of Medicine Committee on the Review of Omics-Based Tests for Predicting Patient Outcomes in Clinical Trials defines omics as the study of related sets of biological molecules in a comprehensive fashion. Omics-based tests are defined as “an assay composed of, or derived from, multiple molecular measurements and interpreted by a fully specified computational model to produce a clinically actionable result” [148].

### 3.2. Biomarker Development

To be used for diagnostics or drug development, an ideal biomarker needs to be highly specific and sensitive [147]. Biomarkers can be classified as pharmacodynamic by indicating the outcome of the interaction between a drug and a target [149] or as prognostic/predictive by stratifying the patient population to responders and non-responders [150]. Another classification that includes three types has been suggested by the Biomarkers and Surrogate End Point Working Group [146,151]: type 0 biomarkers indicate the natural history of disease and correlate with clinical indices, type I biomarkers track the effects of intervention associated with the drug mechanism of action, and type II biomarkers are surrogate end points that predict clinical benefit.

**Figure 3:**
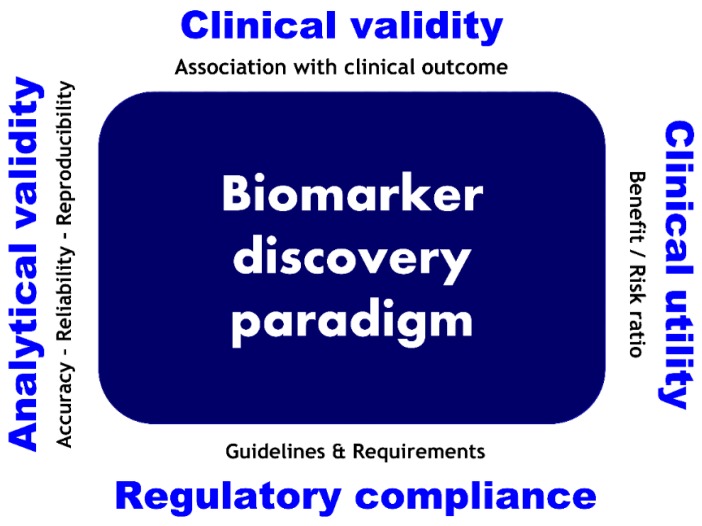
Biomarker development pipeline milestones.

Biomarker development and translational strategies have four main issues that need to be addressed: analytical validity, clinical validity, clinical utility, and regulatory and ethical compliance. Analytical validity includes evidence of assay accuracy, reliability, and reproducibility. Clinical validity denotes evidence regarding the statistical association of biomarkers with the clinical outcome. Clinical utility assesses the benefit of the biomarker in terms of public health. Regulatory and ethical issues address guidelines and requirements compliance of the previous development steps with regulatory bodies and societal challenges, respectively [152]. Figure 3 represents the different pillars of a biomarker discovery pipeline.

### 3.3. Criteria for Omics-Based Biomarkers in Clinical Context

**Figure 4:**
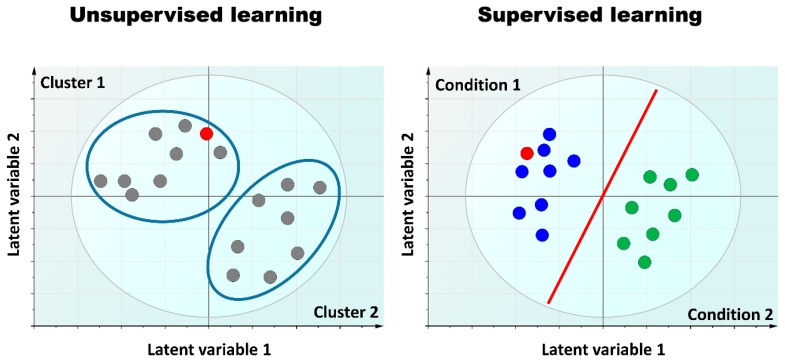
Illustration of the two main machine learning techniques on which omics-based biomarker strategies rely. **Left**: All samples are unlabeled in unsupervised learning. A model separates samples into different clusters based on their biological similarity. A new sample (red circle) is classified according to its similarity to a particular cluster; **Right**: In supervised learning, a training dataset of samples with known class labels is used to build a model (blue circle for condition 1 and green circle for condition 2). The model maximizes the difference between samples from condition 1 and condition 2. Based on this learning, a label for a new sample (red circle) is determined.

Three main aspects entail omics-based test development: analytical development, computational modeling of the predictor, and its clinical utility validation. Given the multi-dimensional and rich information generated by omics data, mathematical modeling is the key to building classifiers for effective medical decision-making. Because omics data are high-dimensional, machine learning and chemometric methods are needed to obtain insights from the data [91]. These methods may be divided into two main classes: unsupervised and supervised methods [76]. Unsupervised methods are exploratory and track patterns in the data; they include principal component analysis [153], independent component analysis [154], k-means clustering [155], hierarchical cluster analysis [156], and self-organizing maps [157]. Supervised methods are mainly predictive and explanatory. They model the dataset so that the class label of separate validation set samples can be predicted based on a series of mathematical models derived from the original data, namely the training set. Different supervised methods such as PLS discriminant analysis (PLS-DA) [158] and orthogonal PLS-DA (OPLS-DA) [159], as well as support vector machines [160], could be applied. For more details, the reader may refer to a recent review [76]. Figure 4 presents a schematic view of the two main computational modeling strategies using machine learning techniques for omics-based biomarker implementation.

**Figure 5:**
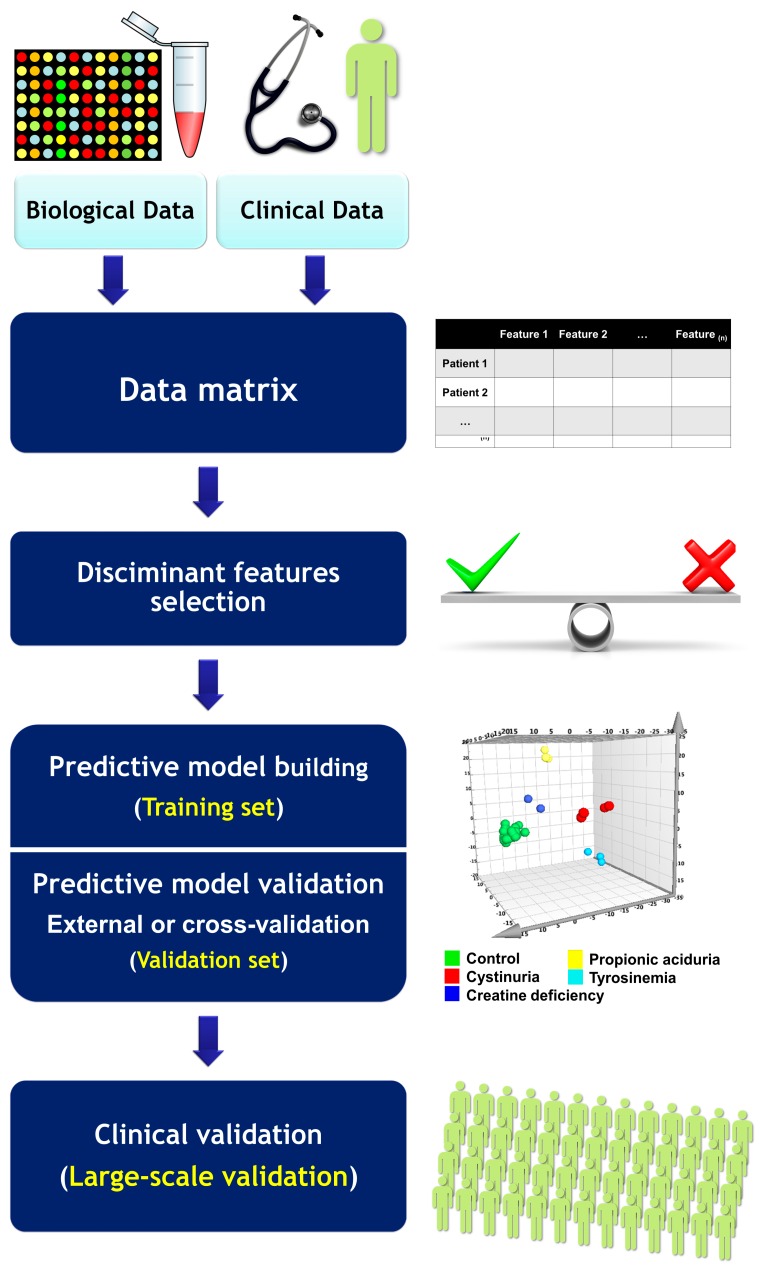
A stepwise approach to using machine learning methods for the prediction of clinical phenotypes. A training dataset is first collected. Then, a subset of features associated with the phenotype of interest is selected. Based on these features, a multi-variate model is built by the training data. A validation set acquired using the same omics profiling methods is collected and treated as new input to the established multi-variate model. The predictions provided by the model are used to assess the classification performance of the test input by comparing the model output and the actual clinical phenotypes of the patients in the validation set.

The high-dimensionality characteristic of omics data requires new approaches for omics-based biomarkers development. McShane et al. described the main issues to take into account during omics-based biomarker development, including samples, analytical development of assays, computational model development, clinical utility assessment, and ethical and regulatory issues. The authors suggested criteria that should be assessed for effective biomarker validation [161]. All these steps raise specific challenges regarding validation practices and determine the use of these omics-based tests [100]. A stepwise approach of using machine learning methods for clinical phenotypes prediction and omics-based predictor development spanning from data collection to large-scale clinical validation are presented in Figure 5.

### 3.4. Omics Integration and the Curse of Dimensionality

Biomedical data are becoming quantitatively (number of samples) and qualitatively (data heterogeneity) complex. The number of samples is driven by the ever-growing high throughput of data acquisition technologies and their digitization, whereas heterogeneity entails biological features (biomolecules, diseases) and related metadata (sampling metadata and clinical data). Furthermore, data could be acquired through different platforms, thus adding bias, complexity, and noise. For these issues, machine learning methods are suitable for data modeling and integration [162]. Data integrative methods can holistically analyze multiple data types to provide systems-level biological insights [91]. Dimensionality reduction techniques have been widely used to handle the biomedical big data deluge, but on a large scale they are computationally intensive. To handle these issues, topological data analysis (TDA) methods may help. TDA methods have emerged recently, but the concept goes back to Leonhard Euler and his work with algebraic topology in the 16th century. TDA methods acquire insight from data by analyzing their shapes (patterns) with geometric dimensional conversions [163,164,165]. These methods have shown good performance in finding hidden patterns when other standard methods fail [95,163,166]. Parsimony phylogenetic analysis is another promising method to handle the omics data deluge [167]. Disease subtype classification for patient stratification is both data-dependent and method-dependent. Thus, it is urgent to have a representative and consistent reference dataset that can be used for the comparison and evaluation of methods.

## 4. Perspectives and Challenges in Translational and Clinical Contexts

### 4.1. Data Integrity, Standardization, and Sharing

Data quality, integrity, and security are the keys to retrieving and maintaining the flow of data and are essential for achieving the promise of “precise” medicine. Data sharing can allow a study to proceed despite the low number of participants, which is often the case in IEM studies. However, the key drivers of data sharing are data and meta-data standards. These are essential for successful data integration and exchange. The lack of such standards or their inconsistent use, especially in omics, are the main drawbacks [102]. Furthermore, in addition to global harmonization, new adapted regulatory approaches for these new omics strategies are urgently needed [168,169].

Large amounts of acquired data raise complex challenges for healthcare stakeholders, including patients. These challenges include the following: (i) sample collection, handling, storage, and transport; (ii) data analyses using multi-omics integration techniques; and (iii) collecting electronic medical record data. The integration of medical record data with biological data and their analysis are other issues. Finally, data sharing within the scientific community raises controversial legal, ethical, and privacy concerns as well [170,171].

### 4.2. Turning Data into Knowledge

Although molecular biomarkers have helped to unveil the underlying pathophysiological mechanisms of disease, only a few of the currently known biomarkers are clinically actionable [172]. When introducing a biomarker to the clinic, it is important to consider its functional characterization through pathways and network analysis, along with its implementation feasibility in terms of public health. Despite the progress in patient phenotyping and stratification, new methods are needed to address the PM era challenges, including analyses of large data [173], integration of multi-type data [174], and simulation of disease behaviors across multi-scale modeling in space and time [91,175,176,177].

### 4.3. Clinical Research Enterprise and Embracing Multi-Disciplinary Sciences

The new omics revolution will play a central role in the post-genomics era of healthcare. To achieve this promise, it is necessary to combine expertise from multiple disciplines, including clinicians, medical laboratory professionals, data scientists, computational biologists, biostatisticians, and lawyers. This observation increases the necessity for new PM teams with new skill sets to develop overlapping expertise for more effective medical interactions across all healthcare partners. Hence, the skill sets of medical professionals need to be diverse; clinical, biological, and computational knowledge to achieve the promises of PM. Training the new generation of the medical workforce to manage and interpret omics data is one solution, and inception of such thinking has already started [178,179,180]. Clinical bioinformatics provides a bridge between omics sciences and clinical practices [181]. We are facing an urgent need to transform all aspects of the healthcare system.

### 4.4. Informatics and New Pathways to Clinical Actionability

Informatics research and innovation are key drivers of the science underlying PM [181]. Actionable biomarkers that aid in clinical decision-making will be envisioned by new frameworks to navigate multi-level evidence regarding whether and how a detected molecular abnormality might be a clinically relevant biomarker [11]. Thanks to databases, accurate annotations with contextual and actionable clinical information will enable the emergence of decision support systems to provide intuitive and patient-specific actionable reports [87,182,183]. Urgent areas to be addressed by clinical bioinformatics research may include biomarker discovery, computational phenotyping, and frameworks for evaluating clinical actionability and utility [181,184,185]. Furthermore, standardization and harmonization-related barriers might trap interoperability and integration by making data aggregation a challenging task [87].

## 5. Paradigm Shift in IEM Investigations

Because IEM are linked to a genetic defect, their current characterization addresses the mutated gene and its products. However, genotype–phenotype correlation is lacking in several IEM, which leads to consideration of the influence of genetic or environmental modifying factors and the impact of an altered pathway on metabolic flux as a whole. These diseases are related to the disruption of specific interactions in a highly organized metabolic network [91,186]. Thus, the impact of a given disruption is not easily predictable [6,187]. Therefore, a functional overview integrating both space and time dimensions is needed to assess the actors of the altered pathway and the potential interactions of each actor [4]. Systemic approaches may address IEM complexity and allow their diagnosis [10,91]. The effectiveness of such approaches has been recently illustrated by van Karnebeek et al. These authors observed a disruption of the *N*-acetylneuraminic acid pathway in patients with severe developmental delay and skeletal dysplasia by using both genomics and metabolomics approaches. As a result, variations in the *NANS* gene encoding the synthase for *N*-acetylneuraminic acid have been identified [10].

**Figure 6:**
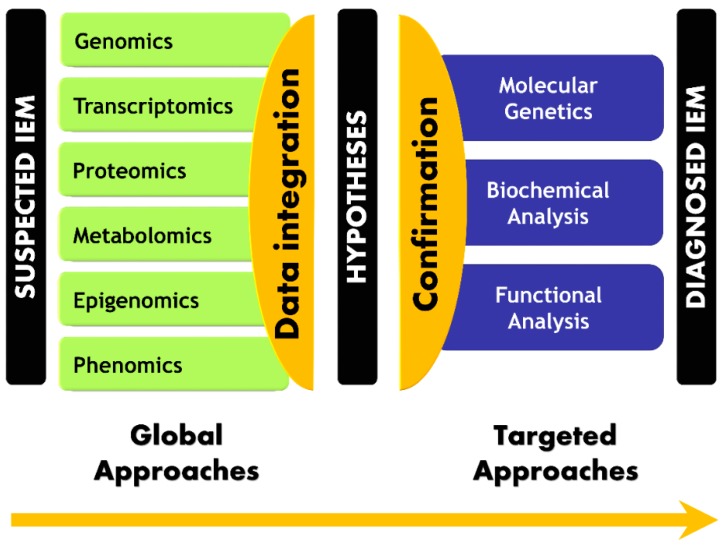
Paradigm shift in Inborn Errors of Metabolism (IEM) diagnosis workflow. Laboratory workflow using high-throughput analytical technologies, integrative bioinformatics, and computational frameworks recovers molecular information for more effective medical decision-making.

Omics-generated data and clinical data integration allow a paradigm shift in IEM handling. An innovative global approach that involves extracting the useful and actionable information may change screening and diagnosis practices. Therefore, a disruptive move from sequential and hypothesis-driven approaches to a global and hypothesis-generating approach is mandatory to embrace the PM era. The core idea of the paradigm shift in the IEM diagnosis workflow is presented in Figure 6.

## 6. Conclusions

Current medical practice is being undermined and PM is profoundly reshaping the future of medicine through recent technological advances. Omics technologies are enabling the simultaneous measurement of a huge number of biochemical entities, including genes, genes expressions, proteins, and metabolites. After decades of reductionism, holistic approaches have begun to address inborn errors of metabolism in a systemic fashion [9,64,91]. Despite some existing drawbacks, genomics and metabolomics seem to be taking the lead in the race to get into clinical practice. However, challenges such as data quality/integrity, reproducibility, and study sample sizes have to be addressed. The small number of multi-omics datasets in the field of IEM and the lack of standardized and harmonized protocols affect the wide dissemination of these approaches. To overcome these drawbacks, attention should be given to validation strategies at all stages. Moreover, the development of new analytical and machine learning methods will facilitate analysis of multi-tissue and multi-organ data, thus enabling a real investigation of systemic effects [95,141,163]. Extended and effective resources for biobanking are also essential to ensure consistency. Addressing these challenges will improve healthcare management of IEM by moving from a reactive, targeted, and reductionist approach to a more proactive, global, and integrative one.

Upgrading laboratory informatics infrastructures and a new medical workforce trained in biomedical big data management are necessary for the successful integration of omics-based strategies. However, the potential of these strategies in the investigation of IEM has yet to be unveiled to all IEM stakeholders worldwide. Laboratory workflows with high-quality data acquisition, mining, and visualization are fundamental for fully embracing the four Ps (predictive, personalized, preventive, and participatory) of PM [188] and effectively translating the underlying biological knowledge into clinically actionable tools.

## Abbreviations

ATAC-seq	Assay for transposase-accessible chromatin next-generation sequencing
BAM	Binary alignment map
ChIP-seq	Chromatin immunoprecipitation next-generation sequencing
CT	Computerized tomography
DNA	Deoxyribonucleic acid
DNase-seq	DNase I digestion of chromatin combined with next-generation sequencing
FDA	Food and Drug Administration
HTS	High-throughput sequencing
ICA	Independent component analysis
IEM	Inborn errors of metabolism
iPF	Integrative phenotyping framework
miRNA	microRNA
ML	Machine learning
MRI	Magnetic resonance imaging
MS	Mass spectrometry
MS/MS	Tandem mass spectrometry
ncRNA	Non-coding RNA
NGS	Next-generation sequencing
OPLSDA	Orthogonal partial least squares discriminant analysis
PCA	Principal component analysis
PLSDA	Partial least squares discriminant analysis
PM	Precision medicine
QC	Quality control
RNA	Ribonucleic acid
rRNA	Ribosome RNA
SAM	Sequence alignment map
SNP	Single-nucleotide polymorphisms
SOM	Self-organizing maps
SOP	Standard operating procedure
SVM	Support vector machines
TDA	Topological data analysis
tRNA	Transfer RNA
VCF	Variant call format
WES	Whole-exome sequencing
WGS	Whole-genome sequencing

## References

[1] Collins F.S., Varmus H. (2015). A new initiative on precision medicine. N. Engl. J. Med..

[2] Ahn A.C., Tewari M., Poon C.S., Phillips R.S. (2006). The limits of reductionism in medicine: Could systems biology offer an alternative?. PLoS Med..

[3] Van Regenmortel M.H. (2004). Reductionism and complexity in molecular biology: Scientists now have the tools to unravel biological and overcome the limitations of reductionism. EMBO Rep..

[4] Aon M.A. (2014). Complex systems biology of networks: The riddle and the challenge. Systems Biology of Metabolic and Signaling Networks.

[5] Kitano H. (2002). Systems biology: A brief overview. Science.

[6] Lanpher B., Brunetti-Pierri N., Lee B. (2006). Inborn errors of metabolism: The flux from mendelian to complex diseases. Nat. Rev. Genet..

[7] Watson J.D., Crick F.H. (1953). The structure of DNA. Cold Spring Harb. Symp. Quant. Biol..

[8] Goodwin S., McPherson J.D., McCombie W.R. (2016). Coming of age: Ten years of next-generation sequencing technologies. Nat. Rev. Genet..

[9] Yang Y., Muzny D.M., Reid J.G., Bainbridge M.N., Willis A., Ward P.A., Braxton A., Beuten J., Xia F., Niu Z. (2013). Clinical whole-exome sequencing for the diagnosis of mendelian disorders. N. Engl. J. Med..

[10] Van Karnebeek C.D., Bonafe L., Wen X.Y., Tarailo-Graovac M., Balzano S., Royer-Bertrand B., Ashikov A., Garavelli L., Mammi I., Turolla L. (2016). Nans-mediated synthesis of sialic acid is required for brain and skeletal development. Nat. Genet..

[11] Tarailo-Graovac M., Shyr C., Ross C.J., Horvath G.A., Salvarinova R., Ye X.C., Zhang L.H., Bhavsar A.P., Lee J.J., Drogemoller B.I. (2016). Exome sequencing and the management of neurometabolic disorders. N. Engl. J. Med..

[12] Worthey E.A., Mayer A.N., Syverson G.D., Helbling D., Bonacci B.B., Decker B., Serpe J.M., Dasu T., Tschannen M.R., Veith R.L. (2011). Making a definitive diagnosis: Successful clinical application of whole exome sequencing in a child with intractable inflammatory bowel disease. Genet. Med..

[13] Benson M. (2016). Clinical implications of omics and systems medicine: Focus on predictive and individualized treatment. J. Intern. Med..

[14] Yohe S., Hauge A., Bunjer K., Kemmer T., Bower M., Schomaker M., Onsongo G., Wilson J., Erdmann J., Zhou Y. (2015). Clinical validation of targeted next-generation sequencing for inherited disorders. Arch. Pathol. Lab. Med..

[15] Yubero D., Brandi N., Ormazabal A., Garcia-Cazorla A., Perez-Duenas B., Campistol J., Ribes A., Palau F., Artuch R., Armstrong J. (2016). Targeted next generation sequencing in patients with inborn errors of metabolism. PLoS ONE.

[16] Cirulli E.T., Goldstein D.B. (2010). Uncovering the roles of rare variants in common disease through whole-genome sequencing. Nat. Rev. Genet..

[17] Stranneheim H., Wedell A. (2016). Exome and genome sequencing: A revolution for the discovery and diagnosis of monogenic disorders. J. Intern. Med..

[18] Meienberg J., Zerjavic K., Keller I., Okoniewski M., Patrignani A., Ludin K., Xu Z., Steinmann B., Carrel T., Rothlisberger B. (2015). New insights into the performance of human whole-exome capture platforms. Nucleic Acids Res..

[19] Mortazavi A., Williams B.A., McCue K., Schaeffer L., Wold B. (2008). Mapping and quantifying mammalian transcriptomes by RNA-Seq. Nat. Methods.

[20] Mensaert K., Denil S., Trooskens G., van Criekinge W., Thas O., de Meyer T. (2014). Next-generation technologies and data analytical approaches for epigenomics. Environ. Mol. Mutagen..

[21] Sanger F., Nicklen S., Coulson A.R. (1977). DNA sequencing with chain-terminating inhibitors. Proc. Natl. Acad. Sci. USA.

[22] Marsh M., Tu O., Dolnik V., Roach D., Solomon N., Bechtol K., Smietana P., Wang L., Li X., Cartwright P. (1997). High-throughput DNA sequencing on a capillary array electrophoresis system. J. Capill. Electrophor..

[23] McBride L.J., Koepf S.M., Gibbs R.A., Salser W., Mayrand P.E., Hunkapiller M.W., Kronick M.N. (1989). Automated DNA sequencing methods involving polymerase chain reaction. Clin. Chem..

[24] Prober J.M., Trainor G.L., Dam R.J., Hobbs F.W., Robertson C.W., Zagursky R.J., Cocuzza A.J., Jensen M.A., Baumeister K. (1987). A system for rapid DNA sequencing with fluorescent chain-terminating dideoxynucleotides. Science.

[25] Venter J.C., Adams M.D., Myers E.W., Li P.W., Mural R.J., Sutton G.G., Smith H.O., Yandell M., Evans C.A., Holt R.A. (2001). The sequence of the human genome. Science.

[26] Lander E.S., Linton L.M., Birren B., Nusbaum C., Zody M.C., Baldwin J., Devon K., Dewar K., Doyle M., FitzHugh W. (2001). Initial sequencing and analysis of the human genome. Nature.

[27] Reuter J.A., Spacek D.V., Snyder M.P. (2015). High-throughput sequencing technologies. Mol. Cell.

[28] Head S.R., Komori H.K., LaMere S.A., Whisenant T., van Nieuwerburgh F., Salomon D.R., Ordoukhanian P. (2014). Library construction for next-generation sequencing: Overviews and challenges. Biotechniques.

[29] Mardis E.R. (2013). Next-generation sequencing platforms. Annu. Rev. Anal. Chem..

[30] Lim E.C., Brett M., Lai A.H., Lee S.P., Tan E.S., Jamuar S.S., Ng I.S., Tan E.C. (2015). Next-generation sequencing using a pre-designed gene panel for the molecular diagnosis of congenital disorders in pediatric patients. Hum. Genom..

[31] Taylor R.W., Pyle A., Griffin H., Blakely E.L., Duff J., He L., Smertenko T., Alston C.L., Neeve V.C., Best A. (2014). Use of whole-exome sequencing to determine the genetic basis of multiple mitochondrial respiratory chain complex deficiencies. JAMA.

[32] Howard H.C., Knoppers B.M., Cornel M.C., Wright Clayton E., Senecal K., Borry P. (2015). Whole-genome sequencing in newborn screening? A statement on the continued importance of targeted approaches in newborn screening programmes. Eur. J. Hum. Genet..

[33] Ashley E.A. (2016). Towards precision medicine. Nat. Rev. Genet..

[34] Goldfeder R.L., Ashley E.A. (2016). A precision metric for clinical genome sequencing. bioRxiv.

[35] Bird A. (2007). Perceptions of epigenetics. Nature.

[36] Huang B., Jiang C., Zhang R. (2014). Epigenetics: The language of the cell?. Epigenomics.

[37] Sadakierska-Chudy A., Filip M. (2015). A comprehensive view of the epigenetic landscape. Part II: Histone post-translational modification, nucleosome level, and chromatin regulation by ncRNAs. Neurotox. Res..

[38] Sadakierska-Chudy A., Kostrzewa R.M., Filip M. (2015). A comprehensive view of the epigenetic landscape part I: DNA methylation, passive and active DNA demethylation pathways and histone variants. Neurotox. Res..

[39] Kundaje A., Meuleman W., Ernst J., Bilenky M., Yen A., Heravi-Moussavi A., Kheradpour P., Zhang Z., Wang J., Ziller M.J. (2015). Integrative analysis of 111 reference human epigenomes. Nature.

[40] Barski A., Cuddapah S., Cui K., Roh T.Y., Schones D.E., Wang Z., Wei G., Chepelev I., Zhao K. (2007). High-resolution profiling of histone methylations in the human genome. Cell.

[41] Yaragatti M., Basilico C., Dailey L. (2008). Identification of active transcriptional regulatory modules by the functional assay of DNA from nucleosome-free regions. Genome Res..

[42] Lister R., O’Malley R.C., Tonti-Filippini J., Gregory B.D., Berry C.C., Millar A.H., Ecker J.R. (2008). Highly integrated single-base resolution maps of the epigenome in arabidopsis. Cell.

[43] Buenrostro J.D., Wu B., Chang H.Y., Greenleaf W.J. (2015). Atac-seq: A method for assaying chromatin accessibility genome-wide. Curr. Protoc. Mol. Biol..

[44] Meyer C.A., Liu X.S. (2014). Identifying and mitigating bias in next-generation sequencing methods for chromatin biology. Nat. Rev. Genet..

[45] Guay S.P., Voisin G., Brisson D., Munger J., Lamarche B., Gaudet D., Bouchard L. (2012). Epigenome-wide analysis in familial hypercholesterolemia identified new loci associated with high-density lipoprotein cholesterol concentration. Epigenomics.

[46] Wang Z., Gerstein M., Snyder M. (2009). RNA-seq: A revolutionary tool for transcriptomics. Nat. Rev. Genet..

[47] Nogales-Gadea G., Consuegra-Garcia I., Rubio J.C., Arenas J., Cuadros M., Camara Y., Torres-Torronteras J., Fiuza-Luces C., Lucia A., Martin M.A. (2012). A transcriptomic approach to search for novel phenotypic regulators in mcardle disease. PLoS ONE.

[48] Mazzoccoli G., Tomanin R., Mazza T., D’Avanzo F., Salvalaio M., Rigon L., Zanetti A., Pazienza V., Francavilla M., Giuliani F. (2013). Circadian transcriptome analysis in human fibroblasts from hunter syndrome and impact of iduronate-2-sulfatase treatment. BMC Med. Genom..

[49] Tringham M., Kurko J., Tanner L., Tuikkala J., Nevalainen O.S., Niinikoski H., Nanto-Salonen K., Hietala M., Simell O., Mykkanen J. (2012). Exploring the transcriptomic variation caused by the finnish founder mutation of lysinuric protein intolerance (LPI). Mol. Genet. Metab..

[50] Dauphinot L., Mockel L., Cahu J., Jinnah H.A., Ledroit M., Potier M.C., Ceballos-Picot I. (2014). Transcriptomic approach to Lesch–Nyhan disease. Nucleosides Nucleotides Nucleic Acids.

[51] Cluzeau C.V., Watkins-Chow D.E., Fu R., Borate B., Yanjanin N., Dail M.K., Davidson C.D., Walkley S.U., Ory D.S., Wassif C.A. (2012). Microarray expression analysis and identification of serum biomarkers for niemann-pick disease, type c1. Hum. Mol. Genet..

[52] Cajka T., Fiehn O. (2015). Toward merging untargeted and targeted methods in mass spectrometry-based metabolomics and lipidomics. Anal. Chem..

[53] Scherl A. (2015). Clinical protein mass spectrometry. Methods.

[54] Kusebauch U., Campbell D.S., Deutsch E.W., Chu C.S., Spicer D.A., Brusniak M.-Y., Slagel J., Sun Z., Stevens J., Grimes B. (2016). Human srmatlas: A resource of targeted assays to quantify the complete human proteome. Cell.

[55] May J.C., McLean J.A. (2016). Advanced multidimensional separations in mass spectrometry: Navigating the big data deluge. Annu. Rev. Anal. Chem..

[56] Tebani A., Schmitz-Afonso I., Rutledge D.N., Gonzalez B.J., Bekri S., Afonso C. (2016). Optimization of a liquid chromatography ion mobility-mass spectrometry method for untargeted metabolomics using experimental design and multivariate data analysis. Anal. Chim. Acta.

[57] James P. (1997). Protein identification in the post-genome era: The rapid rise of proteomics. Quart. Rev. Biophys..

[58] Khoury G.A., Baliban R.C., Floudas C.A. (2011). Proteome-wide post-translational modification statistics: Frequency analysis and curation of the swiss-prot database. Sci. Rep..

[59] Betzen C., Alhamdani M.S.S., Lueong S., Schröder C., Stang A., Hoheisel J.D. (2015). Clinical proteomics: Promises, challenges and limitations of affinity arrays. Proteom. Clin. Appl..

[60] Sabbagh B., Mindt S., Neumaier M., Findeisen P. (2016). Clinical applications of ms-based protein quantification. Proteom. Clin. Appl..

[61] Lassman M.E., McAvoy T., Chappell D.L., Lee A.Y., Zhao X.X., Laterza O.F. (2016). The clinical utility of mass spectrometry based protein assays. Clin. Chim. Acta.

[62] Kovacevic L., Lu H., Goldfarb D.S., Lakshmanan Y., Caruso J.A. (2015). Urine proteomic analysis in cystinuric children with renal stones. J. Pediatr. Urol..

[63] Heywood W.E., Camuzeaux S., Doykov I., Patel N., Preece R.L., Footitt E., Cleary M., Clayton P., Grunewald S., Abulhoul L. (2015). Proteomic discovery and development of a multiplexed targeted mrm-lc-ms/ms assay for urine biomarkers of extracellular matrix disruption in mucopolysaccharidoses I, II, and VI. Anal. Chem..

[64] Williams E.G., Wu Y., Jha P., Dubuis S., Blattmann P., Argmann C.A., Houten S.M., Amariuta T., Wolski W., Zamboni N. (2016). Systems proteomics of liver mitochondria function. Science.

[65] Martens L. (2013). Bringing proteomics into the clinic: The need for the field to finally take itself seriously. Proteom. Clin. Appl..

[66] Holmes E., Wilson I.D., Nicholson J.K. (2008). Metabolic phenotyping in health and disease. Cell.

[67] Oliver S.G., Winson M.K., Kell D.B., Baganz F. (1998). Systematic functional analysis of the yeast genome. Trends Biotechnol..

[68] Nicholson J.K., Lindon J.C., Holmes E. (1999). “Metabonomics”: Understanding the metabolic responses of living systems to pathophysiological stimuli via multivariate statistical analysis of biological NMR spectroscopic data. Xenobiotica.

[69] Nicholson J.K., Holmes E., Kinross J.M., Darzi A.W., Takats Z., Lindon J.C. (2012). Metabolic phenotyping in clinical and surgical environments. Nature.

[70] Suhre K., Raffler J., Kastenmüller G. (2016). Biochemical insights from population studies with genetics and metabolomics. Arch. Biochem. Biophys..

[71] Alonso A., Marsal S., Julia A. (2015). Analytical methods in untargeted metabolomics: State of the art in 2015. Front. Bioeng. Biotechnol..

[72] Therrell B.L., Padilla C.D., Loeber J.G., Kneisser I., Saadallah A., Borrajo G.J., Adams J. (2015). Current status of newborn screening worldwide: 2015. Semin. Perinatol..

[73] Denes J., Szabo E., Robinette S.L., Szatmari I., Szonyi L., Kreuder J.G., Rauterberg E.W., Takats Z. (2012). Metabonomics of newborn screening dried blood spot samples: A novel approach in the screening and diagnostics of inborn errors of metabolism. Anal. Chem..

[74] Aygen S., Durr U., Hegele P., Kunig J., Spraul M., Schafer H., Krings D., Cannet C., Fang F., Schutz B. (2014). NMR-based screening for inborn errors of metabolism: Initial results from a study on turkish neonates. JIMD Rep..

[75] Miller M., Kennedy A., Eckhart A., Burrage L., Wulff J., Miller L.D., Milburn M., Ryals J., Beaudet A., Sun Q. (2015). Untargeted metabolomic analysis for the clinical screening of inborn errors of metabolism. J. Inherit. Metab. Dis..

[76] Tebani A., Abily-Donval L., Afonso C., Marret S., Bekri S. (2016). Clinical metabolomics: The new metabolic window for inborn errors of metabolism investigations in the post-genomic era. Int. J. Mol. Sci..

[77] Houle D., Govindaraju D.R., Omholt S. (2010). Phenomics: The next challenge. Nat. Rev. Genet..

[78] Plomin R., Haworth C.M., Davis O.S. (2009). Common disorders are quantitative traits. Nat. Rev. Genet..

[79] Bush W.S., Oetjens M.T., Crawford D.C. (2016). Unravelling the human genome-phenome relationship using phenome-wide association studies. Nat. Rev. Genet..

[80] Bilder R.M., Sabb F.W., Cannon T.D., London E.D., Jentsch J.D., Parker D.S., Poldrack R.A., Evans C., Freimer N.B. (2009). Phenomics: The systematic study of phenotypes on a genome-wide scale. Neuroscience.

[81] Freimer N., Sabatti C. (2003). The human phenome project. Nat. Genet..

[82] Gerlai R. (2002). Phenomics: Fiction or the future?. Trends Neurosci..

[83] Oetting W.S., Robinson P.N., Greenblatt M.S., Cotton R.G., Beck T., Carey J.C., Doelken S.C., Girdea M., Groza T., Hamilton C.M. (2013). Getting ready for the human phenome project: The 2012 forum of the human variome project. Hum. Mutat..

[84] Groza T., Kohler S., Moldenhauer D., Vasilevsky N., Baynam G., Zemojtel T., Schriml L.M., Kibbe W.A., Schofield P.N., Beck T. (2015). The human phenotype ontology: Semantic unification of common and rare disease. Am. J. Hum. Genet..

[85] Ritchie M.D., Holzinger E.R., Li R., Pendergrass S.A., Kim D. (2015). Methods of integrating data to uncover genotype-phenotype interactions. Nat. Rev. Genet..

[86] Tracy R.P. (2008). “Deep phenotyping”: Characterizing populations in the era of genomics and systems biology. Curr. Opin. Lipidol..

[87] Shameer K., Badgeley M.A., Miotto R., Glicksberg B.S., Morgan J.W., Dudley J.T. (2016). Translational bioinformatics in the era of real-time biomedical, health care and wellness data streams. Brief. Bioinform..

[88] Kochinke K., Zweier C., Nijhof B., Fenckova M., Cizek P., Honti F., Keerthikumar S., Oortveld Merel A.W., Kleefstra T., Kramer J.M. (2016). Systematic phenomics analysis deconvolutes genes mutated in intellectual disability into biologically coherent modules. Am. J. Hum. Genet..

[89] Kim S., Herazo-Maya J.D., Kang D.D., Juan-Guardela B.M., Tedrow J., Martinez F.J., Sciurba F.C., Tseng G.C., Kaminski N. (2015). Integrative phenotyping framework (iPF): Integrative clustering of multiple omics data identifies novel lung disease subphenotypes. BMC Genom..

[90] Mungall C.J., Washington N.L., Nguyen-Xuan J., Condit C., Smedley D., Kohler S., Groza T., Shefchek K., Hochheiser H., Robinson P.N. (2015). Use of model organism and disease databases to support matchmaking for human disease gene discovery. Hum. Mutat..

[91] Argmann C.A., Houten S.M., Zhu J., Schadt E.E. (2016). A next generation multiscale view of inborn errors of metabolism. Cell Metab..

[92] Gligorijevic V., Przulj N. (2015). Methods for biological data integration: Perspectives and challenges. J. R. Soc. Interface.

[93] Wanichthanarak K., Fahrmann J.F., Grapov D. (2015). Genomic, proteomic, and metabolomic data integration strategies. Biomark. Insights.

[94] Wahl S., Vogt S., Stuckler F., Krumsiek J., Bartel J., Kacprowski T., Schramm K., Carstensen M., Rathmann W., Roden M. (2015). Multi-omic signature of body weight change: Results from a population-based cohort study. BMC Med..

[95] Liu W., Bai X., Liu Y., Wang W., Han J., Wang Q., Xu Y., Zhang C., Zhang S., Li X. (2015). Topologically inferring pathway activity toward precise cancer classification via integrating genomic and metabolomic data: Prostate cancer as a case. Sci. Rep..

[96] Chen R., Mias G.I., Li-Pook-Than J., Jiang L., Lam H.Y.K., Chen R., Miriami E., Karczewski K.J., Hariharan M., Dewey F.E. (2012). Personal omics profiling reveals dynamic molecular and medical phenotypes. Cell.

[97] Bartel J., Krumsiek J., Schramm K., Adamski J., Gieger C., Herder C., Carstensen M., Peters A., Rathmann W., Roden M. (2015). The human blood metabolome-transcriptome interface. PLoS Genet..

[98] Shin S.Y., Fauman E.B., Petersen A.K., Krumsiek J., Santos R., Huang J., Arnold M., Erte I., Forgetta V., Yang T.P. (2014). An atlas of genetic influences on human blood metabolites. Nat. Genet..

[99] Petersen A.K., Zeilinger S., Kastenmuller G., Romisch-Margl W., Brugger M., Peters A., Meisinger C., Strauch K., Hengstenberg C., Pagel P. (2014). Epigenetics meets metabolomics: An epigenome-wide association study with blood serum metabolic traits. Hum. Mol. Genet..

[100] Ioannidis J.P., Khoury M.J. (2011). Improving validation practices in “omics” research. Science.

[101] Kolker E., Ozdemir V., Martens L., Hancock W., Anderson G., Anderson N., Aynacioglu S., Baranova A., Campagna S.R., Chen R. (2014). Toward more transparent and reproducible omics studies through a common metadata checklist and data publications. Omics.

[102] Tenenbaum J.D., Sansone S.A., Haendel M. (2014). A sea of standards for omics data: Sink or swim?. JAMIA.

[103] Chitayat S., Rudan J.F. (2016). Chapter 10—Phenome centers and global harmonization. Metabolic Phenotyping in Personalized and Public Healthcare.

[104] Rocca-Serra P., Salek R.M., Arita M., Correa E., Dayalan S., Gonzalez-Beltran A., Ebbels T., Goodacre R., Hastings J., Haug K. (2016). Data standards can boost metabolomics research, and if there is a will, there is a way. Metabolomics.

[105] Dunn W.B., Wilson I.D., Nicholls A.W., Broadhurst D. (2012). The importance of experimental design and QC samples in large-scale and MS-driven untargeted metabolomic studies of humans. Bioanalysis.

[106] Walzer M., Pernas L.E., Nasso S., Bittremieux W., Nahnsen S., Kelchtermans P., Pichler P., van den Toorn H.W., Staes A., Vandenbussche J. (2014). Qcml: An exchange format for quality control metrics from mass spectrometry experiments. Mol. Cell. Proteom..

[107] Issaq H.J., Waybright T.J., Veenstra T.D. (2011). Cancer biomarker discovery: Opportunities and pitfalls in analytical methods. Electrophoresis.

[108] Jonsson P., Wuolikainen A., Thysell E., Chorell E., Stattin P., Wikstrom P., Antti H. (2015). Constrained randomization and multivariate effect projections improve information extraction and biomarker pattern discovery in metabolomics studies involving dependent samples. Metabolomics.

[109] Scherer A. (2009). Batch Effects and Noise in Microarray Experiments: Sources and Solutions.

[110] Vivian J., Rao A., Nothaft F.A., Ketchum C., Armstrong J., Novak A., Pfeil J., Narkizian J., Deran A.D., Musselman-Brown A. (2016). Rapid and efficient analysis of 20,000 RNA-Seq samples with toil. bioRxiv.

[111] Church D.M., Schneider V.A., Steinberg K.M., Schatz M.C., Quinlan A.R., Chin C.-S., Kitts P.A., Aken B., Marth G.T., Hoffman M.M. (2015). Extending reference assembly models. Genome Biol..

[112] Goldfeder R.L., Priest J.R., Zook J.M., Grove M.E., Waggott D., Wheeler M.T., Salit M., Ashley E.A. (2016). Medical implications of technical accuracy in genome sequencing. Genome Med..

[113] Tewhey R., Bansal V., Torkamani A., Topol E.J., Schork N.J. (2011). The importance of phase information for human genomics. Nat. Rev. Genet..

[114] Zheng G.X., Lau B.T., Schnall-Levin M., Jarosz M., Bell J.M., Hindson C.M., Kyriazopoulou-Panagiotopoulou S., Masquelier D.A., Merrill L., Terry J.M. (2016). Haplotyping germline and cancer genomes with high-throughput linked-read sequencing. Nat. Biotechnol..

[115] Chaisson M.J., Huddleston J., Dennis M.Y., Sudmant P.H., Malig M., Hormozdiari F., Antonacci F., Surti U., Sandstrom R., Boitano M. (2015). Resolving the complexity of the human genome using single-molecule sequencing. Nature.

[116] Foquet M., Samiee K.T., Kong X., Chauduri B.P., Lundquist P.M., Turner S.W., Freudenthal J., Roitman D.B. (2008). Improved fabrication of zero-mode waveguides for single-molecule detection. J. Appl. Phys..

[117] Clarke J., Wu H.-C., Jayasinghe L., Patel A., Reid S., Bayley H. (2009). Continuous base identification for single-molecule nanopore DNA sequencing. Nat. Nanotechnol..

[118] Vinaixa M., Schymanski E.L., Neumann S., Navarro M., Salek R.M., Yanes O. (2016). Mass spectral databases for LC/MS- and GC/MS-based metabolomics: State of the field and future prospects. TrAC Trends Anal. Chem..

[119] Wu L., Candille S.I., Choi Y., Xie D., Jiang L., Li-Pook-Than J., Tang H., Snyder M. (2013). Variation and genetic control of protein abundance in humans. Nature.

[120] Vogel C., Marcotte E.M. (2012). Insights into the regulation of protein abundance from proteomic and transcriptomic analyses. Nat. Rev. Genet..

[121] Bittremieux W., Valkenborg D., Martens L., Laukens K. (2016). Computational quality control tools for mass spectrometry proteomics. Proteomics.

[122] Deutsch E.W., Overall C.M., van Eyk J.E., Baker M.S., Paik Y.-K., Weintraub S.T., Lane L., Martens L., Vandenbrouck Y., Kusebauch U. (2016). Human proteome project mass spectrometry data interpretation guidelines 2.1. J. Proteome Res..

[123] Whiteaker J.R., Zhao L., Anderson L., Paulovich A.G. (2010). An automated and multiplexed method for high throughput peptide immunoaffinity enrichment and multiple reaction monitoring mass spectrometry-based quantification of protein biomarkers. Mol. Cell. Proteom..

[124] Fehniger T.E., Boja E.S., Rodriguez H., Baker M.S., Marko-Varga G. (2014). Four areas of engagement requiring strengthening in modern proteomics today. J. Proteome Res..

[125] Shang J., Zhu F., Vongsangnak W., Tang Y., Zhang W., Shen B. (2014). Evaluation and comparison of multiple aligners for next-generation sequencing data analysis. BioMed Res. Int..

[126] Pabinger S., Dander A., Fischer M., Snajder R., Sperk M., Efremova M., Krabichler B., Speicher M.R., Zschocke J., Trajanoski Z. (2014). A survey of tools for variant analysis of next-generation genome sequencing data. Brief. Bioinform..

[127] Evani U.S., Challis D., Yu J., Jackson A.R., Paithankar S., Bainbridge M.N., Jakkamsetti A., Pham P., Coarfa C., Milosavljevic A. (2012). Atlas2 cloud: A framework for personal genome analysis in the cloud. BMC Genom..

[128] Cibulskis K., Lawrence M.S., Carter S.L., Sivachenko A., Jaffe D., Sougnez C., Gabriel S., Meyerson M., Lander E.S., Getz G. (2013). Sensitive detection of somatic point mutations in impure and heterogeneous cancer samples. Nat. Biotechnol..

[129] Koboldt D.C., Zhang Q., Larson D.E., Shen D., McLellan M.D., Lin L., Miller C.A., Mardis E.R., Ding L., Wilson R.K. (2012). Varscan 2: Somatic mutation and copy number alteration discovery in cancer by exome sequencing. Genome Res..

[130] McKenna A., Hanna M., Banks E., Sivachenko A., Cibulskis K., Kernytsky A., Garimella K., Altshuler D., Gabriel S., Daly M. (2010). The genome analysis toolkit: A mapreduce framework for analyzing next-generation DNA sequencing data. Genome Res..

[131] Spencer D.H., Tyagi M., Vallania F., Bredemeyer A.J., Pfeifer J.D., Mitra R.D., Duncavage E.J. (2014). Performance of common analysis methods for detecting low-frequency single nucleotide variants in targeted next-generation sequence data. J. Mol. Diagn..

[132] Manrai A.K., Funke B.H., Rehm H.L., Olesen M.S., Maron B.A., Szolovits P., Margulies D.M., Loscalzo J., Kohane I.S. (2016). Genetic misdiagnoses and the potential for health disparities. N. Engl. J. Med..

[133] Gullapalli R.R., Desai K.V., Santana-Santos L., Kant J.A., Becich M.J. (2012). Next generation sequencing in clinical medicine: Challenges and lessons for pathology and biomedical informatics. J. Pathol. Inform..

[134] Deutsch E.W. (2012). File formats commonly used in mass spectrometry proteomics. Mol. Cell. Proteom..

[135] Misra B.B., van der Hooft J.J. (2016). Updates in metabolomics tools and resources: 2014–2015. Electrophoresis.

[136] Annesley T., Diamandis E., Bachmann L., Hanash S., Hart B., Javahery R., Singh R., Smith R. (2016). A spectrum of views on clinical mass spectrometry. Clin. Chem..

[137] Lathrop J.T., Jeffery D.A., Shea Y.R., Scholl P.F., Chan M.M. (2016). US food and drug administration perspectives on clinical mass spectrometry. Clin. Chem..

[138] Levin N., Salek R.M., Steinbeck C. (2016). Chapter 11—From databases to big data. Metabolic Phenotyping in Personalized and Public Healthcare.

[139] GTEx Consortium (2015). The genotype-tissue expression (GTEX) pilot analysis: Multitissue gene regulation in humans. Science.

[140] Torell F., Bennett K., Cereghini S., Rannar S., Lundstedt-Enkel K., Moritz T., Haumaitre C., Trygg J., Lundstedt T. (2015). Multi-organ contribution to the metabolic plasma profile using hierarchical modelling. PLoS ONE.

[141] Do K.T., Kastenmüller G., Mook-Kanamori D.O., Yousri N.A., Theis F.J., Suhre K., Krumsiek J. (2015). Network-based approach for analyzing intra- and interfluid metabolite associations in human blood, urine, and saliva. J. Proteome Res..

[142] McGregor K., Bernatsky S., Colmegna I., Hudson M., Pastinen T., Labbe A., Greenwood C.M.T. (2016). An evaluation of methods correcting for cell-type heterogeneity in DNA methylation studies. Genome Biol..

[143] Buettner F., Natarajan K.N., Casale F.P., Proserpio V., Scialdone A., Theis F.J., Teichmann S.A., Marioni J.C., Stegle O. (2015). Computational analysis of cell-to-cell heterogeneity in single-cell RNA-Sequencing data reveals hidden subpopulations of cells. Nat. Biotechnol..

[144] Houseman E.A., Molitor J., Marsit C.J. (2014). Reference-free cell mixture adjustments in analysis of DNA methylation data. Bioinformatics.

[145] Bock C., Farlik M., Sheffield N.C. (2016). Multi-omics of single cells: Strategies and applications. Trends Biotechnol..

[146] Biomarkers Definitions Working Group (2001). Biomarkers and surrogate endpoints: Preferred definitions and conceptual framework. Clin. Pharmacol. Ther..

[147] Halim A.-B. (2011). Biomarkers in Drug Development: A Useful Tool but Discrepant Results May Have a Major Impact.

[148] Micheel C.M., Nass S.J., Omenn G.S. (2012). Evolution of Translational Omics: Lessons Learned and the Path Forward.

[149] Feuerstein G., Dormer C., Ruffolo R., Stiles G., Walsh F., Rutkowski J. (2007). Translational medicine perspectives of biomarkers in drug discovery and development. Part I. Target selection and validation-biomarkers take center stage. Int. Drug Discov..

[150] Brünner N. (2009). What is the difference between “predictive and prognostic biomarkers”? Can you give some examples. Connection.

[151] Frank R., Hargreaves R. (2003). Clinical biomarkers in drug discovery and development. Nat. Rev. Drug Discov..

[152] Horvath A.R., Lord S.J., StJohn A., Sandberg S., Cobbaert C.M., Lorenz S., Monaghan P.J., Verhagen-Kamerbeek W.D.J., Ebert C., Bossuyt P.M.M. (2014). From biomarkers to medical tests: The changing landscape of test evaluation. Clin. Chim. Acta.

[153] Hotelling H. (1933). Analysis of a complex of statistical variables into principal components. J. Educ. Psychol..

[154] Rutledge D.N., Bouveresse D.J.-R. (2013). Independent components analysis with the jade algorithm. TrAC Trends Anal. Chem..

[155] Hartigan J.A., Wong M.A. (1979). Algorithm as 136: A k-means clustering algorithm. J. R. Stat. Soc. Ser. C.

[156] Johnson S.C. (1967). Hierarchical clustering schemes. Psychometrika.

[157] Kohonen T. (1990). The self-organizing map. Proc. IEEE.

[158] Wold S., Sjöström M., Eriksson L. (2001). Pls-regression: A basic tool of chemometrics. Chemom. Intell. Lab. Syst..

[159] Trygg J., Wold S. (2002). Orthogonal projections to latent structures (O-PLS). J. Chemom..

[160] Cortes C., Vapnik V. (1995). Support-vector networks. Mach. Learn..

[161] McShane L.M., Cavenagh M.M., Lively T.G., Eberhard D.A., Bigbee W.L., Williams P.M., Mesirov J.P., Polley M.-Y.C., Kim K.Y., Tricoli J.V. (2013). Criteria for the use of omics-based predictors in clinical trials. Nature.

[162] Satagopam V., Gu W., Eifes S., Gawron P., Ostaszewski M., Gebel S., Barbosa-Silva A., Balling R., Schneider R. (2016). Integration and visualization of translational medicine data for better understanding of human diseases. Big Data.

[163] Offroy M., Duponchel L. (2016). Topological data analysis: A promising big data exploration tool in biology, analytical chemistry and physical chemistry. Anal. Chim. Acta.

[164] Lum P., Singh G., Lehman A., Ishkanov T., Vejdemo-Johansson M., Alagappan M., Carlsson J., Carlsson G. (2013). Extracting insights from the shape of complex data using topology. Sci. Rep..

[165] Carlsson G. (2009). Topology and data. Bull. Am. Math. Soc..

[166] Nielson J.L., Paquette J., Liu A.W., Guandique C.F., Tovar C.A., Inoue T., Irvine K.-A., Gensel J.C., Kloke J., Petrossian T.C. (2015). Topological data analysis for discovery in preclinical spinal cord injury and traumatic brain injury. Nat. Commun..

[167] Salazar J., Amri H., Noursi D., Abu-Asab M. (2015). Computational tools for parsimony phylogenetic analysis of omics data. Omics J. Integr. Biol..

[168] Altman R.B., Khuri N., Salit M., Giacomini K.M. (2015). Unmet needs: Research helps regulators do their jobs. Sci. Transl. Med..

[169] Zerhouni E., Hamburg M. (2016). The need for global regulatory harmonization: A public health imperative. Sci. Transl. Med..

[170] Jiang X., Zhao Y., Wang X., Malin B., Wang S., Ohno-Machado L., Tang H. (2014). A community assessment of privacy preserving techniques for human genomes. BMC Med. Inform. Decis. Mak..

[171] Shoenbill K., Fost N., Tachinardi U., Mendonca E.A. (2014). Genetic data and electronic health records: A discussion of ethical, logistical and technological considerations. J. Am. Med. Inform. Assoc..

[172] Poste G. (2011). Bring on the biomarkers. Nature.

[173] Gligorijevic V., Malod-Dognin N., Przulj N. (2016). Integrative methods for analyzing big data in precision medicine. Proteomics.

[174] Li L., Cheng W.Y., Glicksberg B.S., Gottesman O., Tamler R., Chen R., Bottinger E.P., Dudley J.T. (2015). Identification of type 2 diabetes subgroups through topological analysis of patient similarity. Sci. Transl. Med..

[175] Asai Y., Abe T., Li L., Oka H., Nomura T., Kitano H. (2015). Databases for multilevel biophysiology research available at physiome.jp. Front. Physiol..

[176] Garny A., Cooper J., Hunter P.J. (2010). Toward a VPH/physiome toolkit. Wiley Interdiscip. Rev. Syst. Biol. Med..

[177] Clancy C.E., An G., Cannon W.R., Liu Y., May E.E., Ortoleva P., Popel A.S., Sluka J.P., Su J., Vicini P. (2016). Multiscale modeling in the clinic: Drug design and development. Ann. Biomed. Eng..

[178] Henricks W.H., Karcher D.S., Harrison J.H., Sinard J.H., Riben M.W., Boyer P.J., Plath S., Thompson A., Pantanowitz L. (2016). Pathology informatics essentials for residents: A flexible informatics curriculum linked to accreditation council for graduate medical education milestones. J. Pathol. Inform..

[179] Louis D.N., Feldman M., Carter A.B., Dighe A.S., Pfeifer J.D., Bry L., Almeida J.S., Saltz J., Braun J., Tomaszewski J.E. (2015). Computational pathology: A path ahead. Arch. Pathol. Lab. Med..

[180] Louis D.N., Gerber G.K., Baron J.M., Bry L., Dighe A.S., Getz G., Higgins J.M., Kuo F.C., Lane W.J., Michaelson J.S. (2014). Computational pathology: An emerging definition. Arch. Pathol. Lab. Med..

[181] Sirintrapun S.J., Zehir A., Syed A., Gao J., Schultz N., Cheng D.T. (2016). Translational bioinformatics and clinical research (biomedical) informatics. Clin. Lab. Med..

[182] Miotto R., Li L., Kidd B.A., Dudley J.T. (2016). Deep patient: An unsupervised representation to predict the future of patients from the electronic health records. Sci. Rep..

[183] Soualmia L.F., Lecroq T. (2015). Bioinformatics methods and tools to advance clinical care. Findings from the yearbook 2015 section on bioinformatics and translational informatics. Yearb. Med. Inform..

[184] Tenenbaum J.D., Avillach P., Benham-Hutchins M., Breitenstein M.K., Crowgey E.L., Hoffman M.A., Jiang X., Madhavan S., Mattison J.E., Nagarajan R. (2016). An informatics research agenda to support precision medicine: Seven key areas. JAMIA.

[185] Altman R.B., Prabhu S., Sidow A., Zook J.M., Goldfeder R., Litwack D., Ashley E., Asimenos G., Bustamante C.D., Donigan K. (2016). A research roadmap for next-generation sequencing informatics. Sci. Transl. Med..

[186] Sahoo S., Franzson L., Jonsson J.J., Thiele I. (2012). A compendium of inborn errors of metabolism mapped onto the human metabolic network. Mol. BioSyst..

[187] Cho D.-Y., Kim Y.-A., Przytycka T.M. (2012). Chapter 5: Network biology approach to complex diseases. PLoS Comput. Biol..

[188] Hood L., Balling R., Auffray C. (2012). Revolutionizing medicine in the 21st century through systems approaches. Biotechnol. J..

